# Novel genomic alteration in superficial esophageal squamous cell neoplasms in non-smoker non-drinker females

**DOI:** 10.1038/s41598-021-99790-z

**Published:** 2021-10-11

**Authors:** Yusuke Onozato, Yu Sasaki, Yasuhiko Abe, Hidenori Sato, Makoto Yagi, Naoko Mizumoto, Takashi Kon, Takayuki Sakai, Minami Ito, Matsuki Umehara, Ayumi Koseki, Yoshiyuki Ueno

**Affiliations:** 1 Department of Gastroenterology, Faculty of Medicine, Yamagata University, 2-2-2 Iida-Nishi, Yamagata, 990-9585 Japan; 2 Division of Endoscopy, Yamagata University Hospital, 2-2-2 Iida-Nishi, Yamagata, 990-9585 Japan; 3 Genomic Information Analysis Unit, Department of Genomic Cohort Research, Faculty of Medicine, Yamagata University, 2-2-2 Iida-Nishi, Yamagata, 990-9585 Japan

**Keywords:** Cancer, Gastroenterology

## Abstract

Alcohol consumption and smoking pose a significant risk for esophageal squamous cell neoplasia (ESCN) development in males; however, ESCN is often diagnosed in non-drinking and non-smoking females. The mechanisms underlying these differences remain elusive, and understanding them can potentially identify novel pathways involved in ESCN development. We performed short-read sequencing to identify somatic variants on a cancer panel targeting 409 genes using DNA extracted from the superficial squamous cell carcinoma (ESCC) tissues and adjacent non-neoplastic epithelium (NE), and immunohistochemical staining of the protein encoded by the target gene. All male patients (n = 117) were drinkers or smokers, whereas 45% of the female patients (n = 33) were not. Somatic variants were compared among three age-matched groups: 13 female ESCC patients with smoking and drinking habits (known-risk group, F-KR), 13 female ESCC patients without these habits (unknown-risk group, F-UR), and 27 males with ESCC and smoking and drinking habits (M-KR). In the NE, the frequencies of *CDKN2A* variants were significantly higher in F-UR than in F-KR and M-KR. In both ESCC and NE, p14ARF was significantly overexpressed in F-UR than in the other groups. In conclusion, *CDKN2A* might be important in ESCC development, independent of known risk factors.

## Introduction

In Asian countries, including Japan, more than 80% of esophageal cancers are squamous cell carcinoma (ESCC), unlike in Western countries, where adenocarcinoma is the predominant type. According to epidemiological studies of esophageal cancer in Japan, the ratio of males to females is approximately 6:1, with a high prevalence in males and individuals aged 60 to 70 years^1^. Drinking and smoking are considered important risk factors for ESCC; in particular, flushers with reduced activity of alcohol dehydrogenase 1B (ADH1B) and aldehyde dehydrogenase-2 (ALDH2) have a strong risk^2,3^. In fact, a World Health Organization working group has defined acetaldehyde associated with alcoholic drinks as a Group 1 carcinogen^4^. The *ADH1B* gene is classified into three genotypes: ADH1B*1/1 (low activity), ADH1B*1/2 (medium to high activity), and ADH1B*2/2 (high activity)^5^. The risk of ESCC is 2.7 times higher in ADH1B*1/1 than in ADH1B*2 genotypes (ADH1B*1/2 and ADH1B*2/2)^6^. The *ALDH2* gene has two allelic types: active ALDH2*1 and inactive ALDH2*2. The risk of ESCC is 7.1 times higher in ALDH2*1/2 type individuals than in ALDH2*1/1 type individuals^7^.

However, a steady proportion of females without a history of drinking and smoking also develop ESCC. Although the global incidence of ESCC is estimated to be 2.7 times higher in males than in females, the Eastern African Corridor and Asian esophageal cancer belt, including central China, has similar ESCC incidence rates in males and females, whereas ESCC is reported to be more common in females than in males in 12 countries, including several Northeastern African and Middle Eastern countries^8,9^. Thus, in addition to the typical strong-risk factors, there are other potentially important pathways that lead to carcinogenesis. However, in general, the pathogenesis of ESCC has been investigated primarily in male patients with a history of drinking and smoking. Although a better prognosis has been reported in women after surgery or radiation therapy for ESCC^10,11^, there are no reports on the molecular biological mechanisms of ESCC occurrence with respect to gender or risk factors.

Recent bioinformatics analysis using large amounts of short-read data from next-generation sequencing (NGS) has revealed genetic alterations in ESCC, including tumor protein 53 (*TP53*), cyclin-dependent kinase inhibitor 2A (*CDKN2A*), and phosphatidylinositol-4,5-bisphosphate 3-kinase catalytic subunit alpha (*PIK3CA*), which are the major driver genes of ESCC^12–15^. Genetic abnormalities accumulate during the development, invasion, and metastasis of cancers, including colorectal^16^, liver^17^, pancreatic^18^, bladder^19^, breast^20^, and renal cancer^21^. Similarly, ESCC develops in multiple steps from normal epithelium to basal cell hyperplasia, esophageal squamous cell neoplasia (ESCN), intraepithelial carcinoma, and invasive carcinoma, which involves accumulation of aberrations in genes related to cell cycle, apoptosis, and differentiation^22^. Additionally, alterations have been reported in driver genes such as *TP53* and NOTCH homolog 1 (*NOTCH1*) in ESCN and non-cancerous mucosa adjacent to esophageal cancer^23–25^. However, most of these studies focused on advanced ESCC. Although recent studies have reported genomic alteration of ESCN^26,27^ and normal esophageal epithelium with age^28^, genomic alterations in early ESCC remain largely unclear.

In the present study, to clarify the molecular mechanisms underlying the development of ESCC in females without known risk factors such as alcohol consumption and smoking, we investigated the clinical characteristics and genomic alterations of the esophageal epithelium in patients with superficial ESCC with respect to risk factors.

## Results

### Clinical characteristics of the patients

The 150 patients consisted of more males (78%) than females (22%) (*p* < 0.001, Table 1). The median age of all patients was 72 years (IQR: interquartile range, 65–76.2), with no significant difference between males (71 years, IQR 65–76) and females (75 years, IQR 65–77) (*p* = 0.82). All male patients had a history of drinking or smoking, whereas 15 (45%) of the female patients had no history of drinking or smoking (*p* < 0.001). The incidence of esophageal Lugol-voiding lesions (LVLs) grade A was higher in females (24.2%) than in males (5.1%) (*p* = 0.001). There were no differences in tumor location, pathological type, and depth of invasion between the genders.

**Table 1:** Selected characteristics of the patients in the present study.

	Total	Male	Female	*p*-value^a^
Patients, *n* (%)	150	117 (78)	33 (22)	< 0.001
Age, median (IQR)	72 (65–76.2)	71 (65–76)	75 (65–77)	0.828
Drinkers, *n* (%) Drinkers, *n* (%)	120 (80)	104 (88.9)	16 (48.5)	< 0.001
Smokers, *n* (%)	107 (71.3)	99 (84.6)	8 (24.2)	< 0.001
Non-drinkers, non-smokers, *n* (%)	15 (10)	0 (0)	15 (45.5)	< 0.001
Simultaneous multiple neoplasia, *n* (%)	9 (6.0)	6 (5.1)	3 (9.0)	0.397
**LVLs**				
Grade A, *n* (%)	14 (9.3)	6 (5.1)	8 (24.2)	0.001
Grade B, *n* (%)	62 (41.3)	44 (37.6)	18 (54.5)	
Grade C, *n* (%)	72 (48)	65 (55.5)	7 (21.2)	
Unknown, *n* (%)	2 (1.3)	2 (1.7)	0 (0)	
**Tumor location**				
Ce, *n* (%)	1 (0.6)	1 (0.8)	0 (0)	0.784
Ut, *n* (%)	6 (3.8)	4 (3.3)	2 (5.5)	
Mt, *n* (%)	120 (75.5)	92 (74.8)	28 (77.8)	
Lt, *n* (%)	32 (20.1)	26 (21.1)	6 (16.7)	
Ae, *n* (%)	0 (0)	0 (0)	0 (0)	
**Pathological type**				
ESCC, *n* (%)	89 (56.0)	74 (60.2)	15 (41.7)	0.061
ESCN, *n* (%)	70 (44.0)	49 (39.8)	21 (58.3)	
LGIN, *n* (%)	10 (14.3)	6 (12.2)	4 (19.0)	0.879
HGIN, *n* (%)	20 (28.6)	13 (26.5)	7 (33.3)	
CIS, *n* (%)	40 (57.1)	30 (61.2)	10 (47.6)	
**Depth of invasion**				
EP, *n* (%)	95 (59.7)	70 (56.9)	25 (69.4)	0.241
LPM, *n* (%)	34 (21.4)	29 (23.6)	5 (13.9)	
MM, *n* (%)	17 (10.7)	15 (12.2)	2 (5.6)	
SM, *n* (%)	13 (8.2)	9 (7.3)	4 (11.1)	

Clinical characteristics at the time of endoscopic treatment Values are expressed as the median (IQR) or number (%).

^a^Comparison of the values between male and female patients was performed using the χ2 test or Wilcoxon rank-sum test. Statistical calculations were performed using JMP 14.3.0

*IQR* interquartile range; *LVLs* Lugol-voiding lesions; *Ce* cervical esophagus; *Ut* upper thoracic esophagus; *Mt* middle thoracic esophagus; *Lt* lower thoracic esophagus; *Ae* abdominal esophagus; *ESCC* esophageal squamous cell carcinoma; *ESCN* esophageal squamous cell neoplasia; *LGIN* low-grade intraepithelial neoplasia; *HGIN* high-grade intraepithelial neoplasia; *CIS* carcinoma in situ; *EP* epithelium; *LPM* lamina propria mucosa; *MM* muscularis mucosa; *SM* submucosa.

After excluding the two patients whose drinking or smoking history was unknown, we stratified the patients into three groups: females with no history of drinking or smoking as the female unknown-risk group (F-UR, n = 15), females with a drinking or smoking history as the female known-risk group (F-KR, n = 17), and males with a drinking or smoking history as the male known-risk group (M-KR, n = 116) (Table 2). No significant differences were observed in the median age (*p* = 0.42) and proportion of pathological type (*p* = 0.12) among the three groups. In the F-UR group, all patients presented the ADH1B*2/2 allele type (*p* = 0.002, Suppl. Table 1). The three groups exhibited no difference in the *ALDH2* allele type (*p* = 0.46, Suppl. Table 1). The frequency of LVL according to grade was significantly different among the three groups (*p* < 0.001), with no patients of grade C in the F-UR group and no patients of grade A in the F-KR group. The M-KR group exhibited the highest proportion of patients with grade C disease (56.0%).

**Table 2:** Comparison of the selected characteristics by risk factors.

	Total	Female unknown-risk	Female known-risk	Male known-risk	*p*-value^b^
Patients, *n* (%)	148	15 (10.1)	17 (11.5)	116 (78.4)	< 0.001
Age, median (IQR)	72 (65–76)	76 (65–78)	74 (60–76)	71 (65–76)	0.418
Simultaneous multiple neoplasia, *n* (%)	9 (6.0)	0 (0)	3 (17.6)	6 (5.1)	0.092
**LVLs**^a^					
Grade A, *n* (%)	14 (9.5)	8 (53.3)	0 (0)	6 (5.2)	< 0.001
Grade B, *n* (%)	61 (41.2)	7 (46.7)	10 (58.8)	44 (37.9)	
Grade C, *n* (%)	72 (48.6)	0 (0)	7 (41.2)	65 (56.0)	
Unknown, *n* (%)	1 (0.7)	0 (0)	0 (0)	1 (0.9)	
**Pathological type**					
ESCC, *n* (%)	88 (56.1)	5 (33.3)	10 (50.0)	73 (59.8)	0.125
ESCN, *n* (%)	69 (43.9)	10 (66.7)	10 (50.0)	49 (40.2)	
LGIN, *n* (%)	9 (13.0)	1 (10.0)	2 (20.0)	6 (12.2)	0.398
HGIN, *n* (%)	20 (29.0)	2 (20.0)	5 (50.0)	13 (26.5)	
CIS, *n* (%)	40 (58.0)	7 (70.0)	3 (30.0)	30 (61.2)	

After excluding the two patients whose drinking or smoking history was unknown, the patients were stratified into three groups: female unknown-risk group, female known-risk group, and male known-risk group. Values are expressed as the median (IQR) or number (%).

^a^In one patient, the grade classification of LVLs was difficult to determine from endoscopic images. ^b^Comparisons among the three groups were performed using the χ2 test or ANOVA. Statistical calculations were performed using JMP 14.3.0

*LVLs* Lugol-voiding lesions; *ESCN* esophageal squamous cell neoplasia; *LGIN* low-grade intraepithelial neoplasia; *HGIN* high-grade intraepithelial neoplasia; *CIS* carcinoma in situ; *ESCC* esophageal squamous cell carcinoma; *IQR* interquartile range; *ANOVA* analysis of variance.

### Comparison of somatic alterations between neoplastic and adjacent non-neoplastic epithelium

We attempted to isolate DNA from the formalin-fixed paraffin-embedded (FFPE) tissues of 30 M-KR patients, twice the number of patients in F-UR, who were randomly matched for age with all F-KR and F-UR patients. NGS analysis was not available for 3 patients in M-KR, 3 patients in F-KR, and 2 patients in F-UR because of low DNA quantity or quality. Finally, age-matched patients in the three groups (27 patients in M-KR; 13, F-KR; and 13, F-UR) were subjected to NGS analysis. The clinical characteristics of these selected patients were similar to that of the overall study population, and the proportion of ESCN was higher in the F-UR patients than in the F-KR and M-KR patients (Table 3, Suppl. Table 2). The median total number of variants in the neoplastic epithelium (Suppl. Figure 1) was higher in the M-KR group (4823.5, IQR 2267–8846) than in the F-UR (2976, IQR 1311–6571) and F-KR (1802, IQR 1247–4423) groups; however, the difference was not significant (*p* = 0.09). Additionally, there was no significant difference in the frequency of variant types among the three groups. The median total number of variants in the adjacent non-neoplastic epithelium (Suppl. Figure 2) was significantly different among the F-UR (6499, IQR 3221–8283), M-KR (3826, IQR 2307–5937), and F-KR (2459, IQR 957–4525) groups (*p* = 0.017) and significantly higher in the F-UR group than in the F-KR group (*p* = 0.012). The number of single nucleotide (*p* = 0.004), start-loss (*p* = 0.008), and stop-gain (*p* = 0.011) variants in the adjacent non-neoplastic epithelium were significantly higher in the F-UR group than in the F-KR group.

**Table 3:** Characteristics of patients who underwent NGS analysis in the present study.

	Total	Female unknown risk	Female known risk	Male known risk	*p*-value^a^
Patients, *n* (%)	53	13 (24.5)	13 (24.5)	27 (50.9)	
Age, median (IQR)	74 (65–77)	76 (65–78)	75 (60–76)	74 (64–77)	0.727
**LVLs**					
Grade A, *n* (%)	11 (20.8)	7 (53.8)	0 (0)	4 (14.8)	0.005
Grade B, *n* (%)	29 (54.7)	6 (46.2)	9 (69.2)	14 (51.9)	
Grade C, *n* (%)	13 (24.5)	0 (0)	4 (30.8)	9 (33.3)	
Unknown, *n* (%)	0 (0)	0 (0)	0 (0)	0 (0)	
**Pathological type**					
ESCC, *n* (%)	36 (67.9)	5 (38.5)	8 (61.5)	23 (85.2)	0.010
ESCN, *n* (%)	17 (32.1)	8 (61.5)	5 (38.5)	4 (14.8)	

Values are expressed as median (IQR) or number (%). ^a^Comparisons among the three groups were performed using the χ2 test or ANOVA. Statistical calculations were performed using JMP 14.3.0

*LVLs* Lugol-voiding lesions; *ESCN* esophageal squamous cell neoplasia; *ESCC* esophageal squamous cell carcinoma; *ANOVA* analysis of variance; *IQR* interquartile range.

### Differences in gene variant frequencies

**Figure 1:**
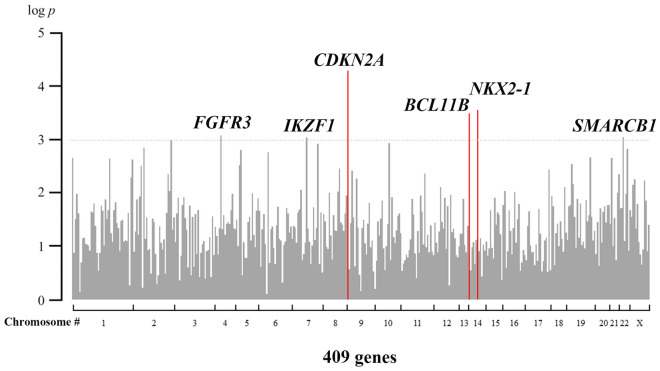
Differences in the frequency of somatic variants by risk factors. The number of variants in 409 genes was compared using ANOVA, with FDR < 0.05, among six groups, including somatic variants in neoplastic and adjacent non-neoplastic epithelium of the F-UR, F-KR, and M-KR groups. There was a significant difference in the frequency of *CDKN2A* (*p* < 0.0001), *NKX2-1* (*p* = 0.0002), and *BCL11B* (*p* = 0.0003) gene variants (*p* < 0.001). Statistical calculations were performed using R programming language version 3.6.1. *F-UR* female unknown-risk; *F-KR* female known-risk; *M-KR* male known-risk; *FDR* false discovery rate; *ANOVA* analysis of variance; *CDKN2A* cyclin-dependent kinase inhibitor 2A; *NKX-21* NKX homebox-1; *BCL11B* B-cell lymphoma/leukemia 11B; *Chr* chromosome.

**Figure 2:**
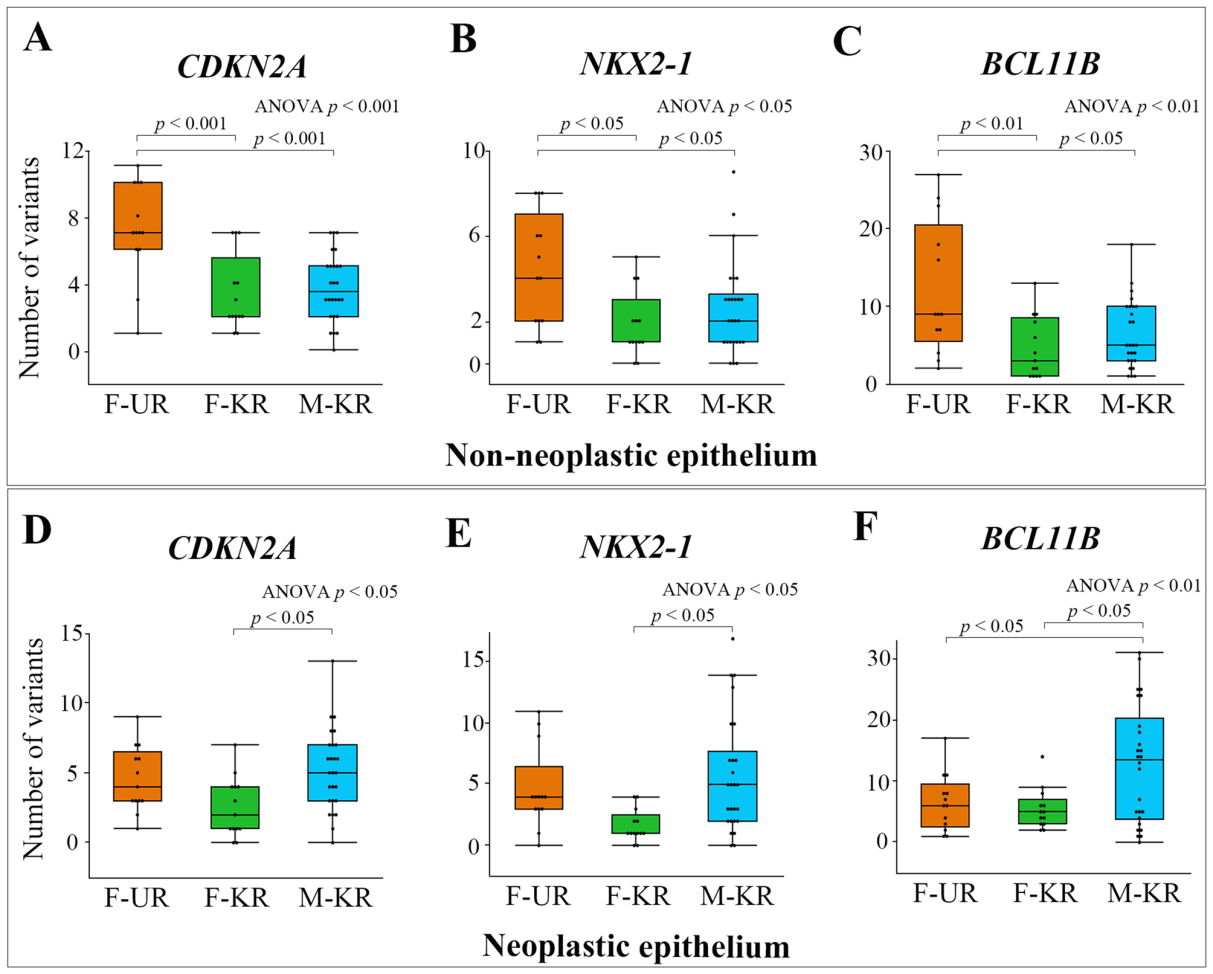
Comparison of the number of somatic variants in neoplastic and the adjacent non-neoplastic epithelium by risk factors. In adjacent non-neoplastic epithelium, the number of somatic variants of the *CDKN2A* (**A**), *NKX2-1* (**B**), and *BCL11B* (**C**) genes were significantly higher in the F-UR group than in the F-KR and M-KR groups. In the neoplastic epithelium, the number of somatic variants of the *CDKN2A* (**D**), *NKX2-1* (**E**), and *BCL11B* (**F**) genes were significantly different between the F-KR and M-KR groups, and the number of *BCL11B* gene variants was significantly higher in the M-KR group than in the F-UR group; however, there was no difference in the number of these three gene somatic variants in F-UR between either the F-KR or M-KR. Box plot: the bottom and top of each box represent the 25th and 75th percentiles, respectively, and the band in the box is the median. Whiskers: the lowest datum is within the minimum, and the highest datum is still within the 1.5 IQR of the upper quartile. We used ANOVA with a post-hoc Tukey–Kramer test to evaluate the statistical differences among the groups. Statistical calculations were performed using JMP 14.3.0. *CDKN2A* cyclin-dependent kinase inhibitor 2A; *NKX-21* NKX homebox-1; *BCL11B* B-cell lymphoma/leukemia 11B; *F-UR* female unknown-risk; *F-KR* female known risk; *M-KR* male known risk; *ANOVA* analysis of variance; *IQR* interquartile range.

The frequency of each gene variant was compared using analysis of variance (ANOVA) among the six groups of neoplastic and adjacent non-neoplastic epithelium of the F-UR, F-KR, and M-KR groups (Fig. 1). The number of gene variants of cyclin-dependent kinase inhibitor 2A (*CDKN2A*), NKX homeobox-1 (*NKX2-1*), and B-cell lymphoma/leukemia 11 B (*BCL11B*) in the adjacent non-neoplastic epithelium were significantly higher in the F-UR than in the F-KR and M-KR groups (Fig. 2A–C). The largest difference was noted for the *CDKN2A* gene variants, which had a higher variant allele frequency (VAF) above 0.9, as well as the *TP53* gene variants (Suppl. Figure 3). In contrast, in the neoplastic epithelium, the number of variants of the three genes in the F-UR group was not different from that in the F-KR and M-KR groups (Fig. 2D–F).

### Comparison of *CDKN2A* variant frequencies in ESCC and ESCN

In the neoplastic epithelium, there was no significant difference in the frequency of *CDKN2A* gene variants among the three groups in both ESCC and ESCN patients (Suppl. Figure 4); however, among the three groups of ESCN patients, the F-UR patients exhibited the highest variant frequency. In the adjacent non-neoplastic epithelium, there was a significant difference in the frequency of *CDKN2A* gene variants among the three groups for both ESCC (*p* = 0.032) and ESCN patients (*p* = 0.007); these values were the highest in the F-UR group.

### Correlation between frequency of *CDKN2A* variants and age

We analyzed the correlation between the frequency of *CDKN2A* gene variants and age in the neoplastic and adjacent non-neoplastic epithelium using Spearman's rank correlation coefficient (Suppl. Figure 5). There was no correlation between the frequency of *CDKN2A* gene variants and age in either neoplastic (r = 0.035) or adjacent non-neoplastic epithelium (r =  − 0.096).

### Expression levels of p16 inhibitor of cyclin-dependent kinase 4A (p16INK4A) and p14 alternate reading frame (p14ARF) in tissues according to risk factors

**Figure 3:**
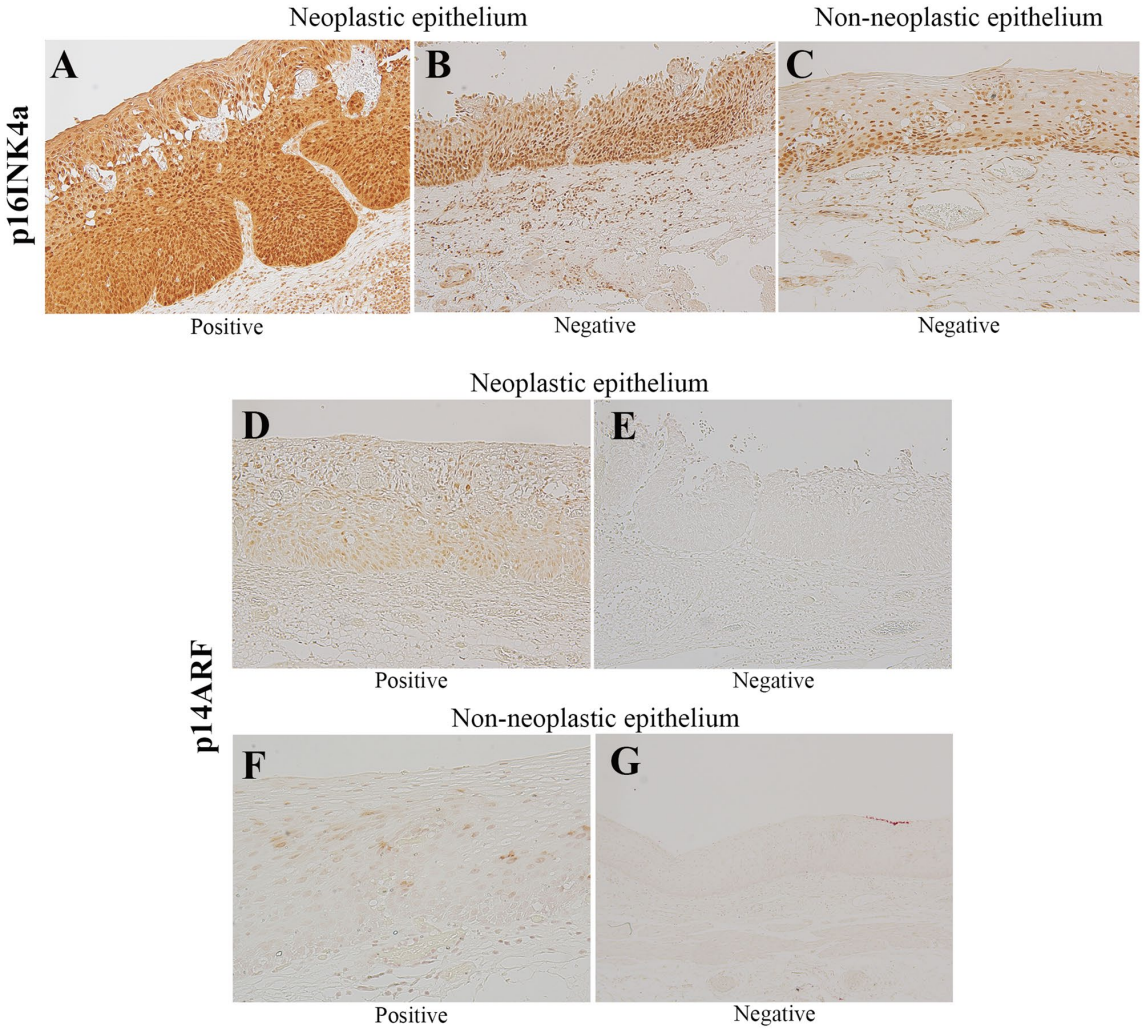
Representative immunohistochemical images of p16INK4a and p14ARF. Both the nucleus and cytoplasm were stained in p16INK4a-positive neoplastic epithelium (**A**); in contrast, in the p16INK4a-negative neoplastic epithelium, the cytoplasm was unstained (**B**). In all patients, no cytoplasmic p16INK4a-positive images were observed for the non-neoplastic epithelium (**C**). The nucleus was stained in p14ARF-positive neoplastic epithelium (**D**); in contrast, in the p14ARF-negative neoplastic epithelium, the nuclei were unstained (**E**). Patients for whom the non-neoplastic epithelium stained for p14ARF in the nucleus were considered positive (**F**), and those for whom this staining was not observed were considered negative (**G**). In both neoplastic and non-neoplastic epithelium, the expression of p16INK4A in the cytoplasm and p14ARF in the nucleus (> 10%) were considered positive. *p14ARF* p14 alternate reading frame; *p16INK4a* p16 inhibitor of cyclin-dependent kinase 4A.

We compared the expression levels of p16INK4a and p14ARF in resected specimens of superficial ESCC (Fig. 3) among the three groups (Table 4). The p14ARF expression levels were significantly different among the three groups in both neoplastic (*p* = 0.04) and adjacent non-neoplastic epithelium (*p* = 0.007) and were the highest in the F-UR group. There was no difference in the expression of p16INK4a in the neoplastic epithelium (*p* = 0.38), and no expression was observed in the non-neoplastic epithelium.

**Table 4:** Expression levels of p14ARF and p16INK4a in neoplastic and non-neoplastic epithelium.

	Neoplastic epithelium			Non-neoplastic epithelium			*p*^a^	*p*^b^
	F-UR (*n* = 13)	F-KR (*n* = 13)	M-KR (*n* = 27)	F-UR (*n* = 13)	F-KR (*n* = 13)	M-KR (*n* = 27)		
p14ARF *n* (%)	5 (38.5)	2 (15.3)	2 (7.4)	3 (23.1)	0 (0)	0 (0)	0.049	0.007
p16INK4a *n* (%)	9 (69.2)	6 (46.2)	18 (66.7)	0 (0)	0 (0)	0 (0)	0.382	-

Values are expressed as the number (%). Comparisons among the three groups of neoplastic^a^ or non-neoplastic epithelium^b^ were performed using the χ2 test. Statistical calculations were performed using JMP 14.3.0

*F-UR* female unknown-risk; *F-KR* female known-risk; *M-KR* male known-risk; *p16INNK4* p16 inhibitor of cyclin-dependent kinase 4A; *p14ARF* p14 alternate reading frame.

## Discussion

In the present study, we showed for the first time that in female patients with ESCC without drinking and smoking habits, which are considered risk factors for ESCC, there are distinctive genetic alterations in the esophageal epithelium, particularly a high frequency of *CDKN2A* gene variants in adjacent non-neoplastic epithelium and high p14ARF expression in neoplastic and adjacent non-neoplastic epithelium.

Among the patients with superficial ESCC, 10% were females with no history of drinking or smoking. In this F-UR group, the incidence of low-risk LVL grade A was high, whereas the incidence of strong-risk grade C was high in the F-KR and M-KR groups. The endoscopic findings indicated that the background epithelium of the F-UR group was different from that of the F-KR and M-KR groups. The risk of ESCC associated with alcohol consumption is known to be increased in ADH1B*1/1 and ALDH2*1/2^6,7^. The alleles of *ALDH2* revealed no difference among the three groups; however, all patients in the F-UR group presented ADH1B*2/2, which is associated with a low risk of esophageal cancer. These findings imply that the development of ESCC in the F-UR group was triggered through a mechanism different from known risk exposures, such as alcohol consumption and smoking.

In general, *TP53* gene variants are the most common genetic alterations observed in ESCC, in addition to the genes histone-Lysine N-methyltransferase 2D, notch homolog 1 (*NOTCH1*), zinc finger protein 750, *PIK3CA*, and E1A binding protein 300^15^. Yokoyama et al. showed that variants in the genes of *TP53*, nuclear factor*,* erythroid 2-like 2, *CDKN2A*, and F-box and WD repeat domain containing 7 were more common in ESCC than in normal esophageal squamous epithelium^25^. In normal esophageal squamous epithelium, variants in *NOTCH1-3*, FAT tumor suppressor homolog 1, zinc finger protein 36 like 2, protein phosphatase*,* Mg^2+^/Mn^2+^ dependent 1D, checkpoint kinase 2, and paired box gene 9 were more common than in ESCC. In particular, in normal esophageal squamous epithelium, the number of clones with *NOTCH1* alterations expands with age and is promoted through alcohol consumption and smoking, contributing to carcinogenesis. In addition, Urabe et al. reported that somatic alterations such as *TP53*, *NOTCH1*, deletion of *CDKN2A*, and amplification of cyclin D1 are more frequent in cancerous mucosa than in non-cancerous mucosa and that these alterations play important roles in esophageal mucosal carcinogenesis^29^. However, all these reports were based on ESCC patients with known risks such as alcohol consumption and smoking. The *CDKN2A* gene is a known driver gene for ESCC; however, there have been no reports on the presence of its somatic variants specifically in the background epithelium of ESCC in females without a history of drinking or smoking.

Unexpectedly, the number of *CDKN2A* variants in the non-neoplastic epithelium in the F-UR group was higher than that in the neoplastic epithelium. Intriguingly, alterations in driver genes such as *NOTCH1* in esophageal cancer have been reported to be more frequent in non-cancerous areas than in cancerous areas in relation to age, suggesting that the mechanisms of clonal growth in the non-cancerous epithelium and esophageal cancer are not fully equivalent and that each has its own unique mechanism^25,28^. In the present study, the frequency of *CDKN2A* variants did not correlate with age, and this factor could not be clarified; however, it may be considered as data supporting these results. In addition, we performed preliminary analysis of the loss of heterozygosity (LOH) and copy number alterations (CNAs) of the region harboring the *CDKN2A* loci. LOH of the region harboring the *CDKN2A* loci was not identified. CNAs of the region harboring the *CDKN2A* loci were detected in 11.3% (6 of 53) of the cases, although the frequency did not differ among the groups (Suppl. Figure 6). Therefore, the CNAs seem to have no significant impact on the highly frequent *CDKN2A* variants in the adjacent non-neoplastic epithelium of the F-UR group. A recent large-scale genome-wide reconstruction of the evolutionary history of cancers^30^ demonstrated that over time, tumors evolve and follow increasingly diverse pathways, driven by individual rare driver mutations and CNAs. However, none of these trends is absolute, and the evolutionary pathways of individual tumors may be very diverse. In this study, we focused on the F-UR group, which was previously assumed to be at low risk for ESCC, and might have uncovered a clue to one of the unique trajectories in ESCC development during somatic evolution.

The *CDKN2A* gene is located at 9p21 and generates two different proteins by selective splicing, p16INK4A and p14ARF, which are involved in cell cycle regulation^31^. Both act as tumor suppressor genes; p16INK4A is involved in the retinoblastoma (Rb) pathway and p14ARF in the p53 pathway^32^. p16INK4A binds directly to the cyclin D/cyclin-dependent kinase 4 complex and activates Rb protein by inhibiting its phosphorylation^32^. Alterations of p16INK4A have been reported in bladder^33^, prostate^34^, kidney^35^, brain^36^, lung^37^, and colon^37^ cancers and leukemia^37^. Furthermore, p16INK4A alterations are observed in head and neck^38^, pancreatic^39^, and colorectal cancer^40^ in precancerous lesions to early stages of the cancer, and the frequency of the alteration increases with the stage and invasiveness of the malignancy^37^. In advanced ESCC, homozygous deletion of p14ARF was observed to occur more frequently than homozygous deletion of p16INK4A; however, p16INK4A genomic alteration was reported to be more common than p14ARF genomic alteration^41^. In the present study, p16INK4A expression in neoplastic and adjacent non-neoplastic epithelium of superficial ESCC and ESCN showed no difference with respect to risk exposure. Although the Rb pathway involving p16INK4A appears to be an important mechanism for ESCC development, it is unlikely to be specifically involved in ESCC carcinogenesis in females without known risk.

In cervical cancer, p16INK4A is a well-known surrogate marker for human papillomavirus (HPV) infection, which is established as a carcinogen. However, the association between HPV infection and ESCC carcinogenesis remains controversial^42^. In the present study, immunohistochemical expression of p16INK4 was not observed in the non-neoplastic epithelium, whereas expression of p16INK4 was observed in 46–69% of neoplastic epithelium. Although there were no differences in the expression according to risk, these findings may raise the possibility of HPV involvement in the development of ESCC.

The expression of p14ARF in both neoplastic and adjacent non-neoplastic epithelium was significantly higher in the F-UR group than in the M-KR and F-KR groups. p14ARF inhibits p53 ubiquitination by trapping murine double minute 2 (MDM2) in the nucleus, thereby stabilizing the tumor suppressor p53. Loss of p14ARF promotes p53 ubiquitination, which in turn promotes carcinogenesis^43^. Alterations in the *p14ARF* gene have been found in glioblastoma^44^, malignant lymphoma^36^, and lung cancer^45^. p14AFR downstream of MDM2 overexpression is directly linked to estrogen receptor (ER) α overexpression^46–48^, and MDM2 mRNA is upregulated in ER-α-positive invasive breast cancer^49^. Estrogen treatment in breast cancer cell lines has been shown to overexpress MDM2 and promote carcinogenesis^50^. Moreover, the expression levels of ERα and ERβ, based on immunohistochemical staining, have been correlated with prognosis in female patients with advanced ESCC, and ERα expression or ERβ non-expression are associated with poor prognosis^51^. In the present study, the expression level of p14ARF in both neoplastic and adjacent non-neoplastic epithelium was higher in female patients without known risks than in patients with known risks. In the background mucosa of female ESCC patients without known risks, p14ARF dysfunction coupled with estrogen-induced enhancement of MDM2 action may promote ESCC carcinogenesis. Although the differences in the proportion of p14ARF expression were statistically significant, the number of cases was limited, and it seems inconclusive whether p14ARF expression is unique to F-UR or not based on the present study alone. We believe that further validation with a larger cohort will be necessary in the future.

Additionally, the *CDKN2A* gene has been associated with cellular senescence^52^. In contrast, it has been reported that p14ARF is not directly involved in human cellular senescence because its expression does not change during cellular senescence^53^. In the present study, there was no significant difference in age among the three groups, and the frequency of *CDKN2A* gene variants did not correlate with age. Therefore, the effect of age on the differences in *CDKN2A* gene variants appeared to be limited in the present study.

The genetic characteristics of ESCC and ESCN might be different. However, genomic analysis of ESCC, adjacent low-grade intraepithelial neoplasia (LGIN), and high-grade intraepithelial neoplasia (HGIN) revealed that the genomic variant profiles of LGIN and HGIN are similar to that of ESCC^26^, including the profile for *TP53* variants. In the present study, there was no obvious difference in the frequency of *CDKN2A* gene variants between superficial ESCC and ESCN, which suggests that alterations in this gene are introduced during the ESCN stage.

This study has several limitations. First, selection bias may exist because of the retrospective nature of the study. Second, the number of patients in the study was limited, which may have affected the conclusion. Third, we could not clarify the factors underlying the unique genomic alterations in female ESCC patients without known risks. It is necessary to identify novel risk factors for ESCC in a large population with detailed clinical information. Fourth, the present study did not include male patients with ESCC who had never consumed alcohol or smoked. Further investigation is needed to determine whether gender differences affect the results of the present study. Fifth, esophageal squamous epithelium from healthy subjects was not available for this analysis because of the difficulty in collecting it owing to ethical reasons. Therefore, it could not be clarified whether the alterations were specific to patients with superficial ESCC. To evaluate that the *CDKN2A* variant is a major clone, analysis of multiple sampling and clonal structure by copy number corrected VAF, which is supposed to approximate cell fractionation, seems to be an intriguing challenge. In addition, further investigation is needed to clarify the precise molecular mechanisms underlying the association between somatic alterations and ESCC development in female patients without known risk.

In the present study, we demonstrated for the first time that *CDKN2A* gene variants were significantly more abundant in the background epithelium of ESCC patients without risk factors such as alcohol consumption and smoking. Moreover, we showed that p14ARF, encoded by the *CDKN2A* gene, was overexpressed in the neoplastic and adjacent non-neoplastic epithelium of these patients. In superficial ESCC, there is a distinctive carcinogenic pathway that is not associated with known risk factors, and the *CDKN2A* gene appears to play an important role in this pathway. These findings should encourage further exploration of the significance of the molecular features of ESCC in the F-UR group and the underlying mechanisms. Patients do exist, albeit in rare cases. To achieve a complete treatment for esophageal cancer, careful evaluation of patients who are not in the limelight, as in this case, will probably be necessary. These efforts will hopefully lead to the establishment of new treatment and diagnostic methods for ESCC patients.

## Methods

### Study participants

In the present study, we included 150 consecutive patients (159 cases) with superficial ESCC, including ESCN, who underwent endoscopic resection at the Yamagata University Hospital between January 2009 and December 2018. ESCN included LGIN, HGIN, and carcinoma in situ (CIS). We obtained clinical information on alcohol consumption, smoking, endoscopic findings, histological type, and depth of invasion from their medical records. We defined drinkers as patients with daily drinking habits and smokers as patients with a current or past habit of smoking at least one cigarette daily. Before endoscopic treatment, blood samples were collected from their veins by a nurse after overnight fasting and immediately frozen at − 80 °C.

The present study was approved by the Ethics Review Committee of Yamagata University Faculty of Medicine (#2018-440) and was conducted in accordance with the Declaration of Helsinki. Written informed consent was obtained from all subjects whose blood samples were collected, in addition to the opt-out on the website (https://www.id.yamagata-u.ac.jp/ethics/rinshou/pdfs/2018/2018-440.pdf).

### Allelic identification of *ADH1B* and *ALDH2* genotypes

Alleles of *ADH1B* and *ALDH2* were identified in patients whose whole blood had been stored (n = 106; 84 males and 22 females). DNA was extracted from the whole blood using the QIAmp DNA Blood Mini Kit (QIAGEN, Hilden, Germany) according to the manufacturer's protocol. The DNA concentrations were quantified using a NanoVue spectrophotometer (GE Healthcare Life Sciences, USA). The genotypes of *ADH1B* and *ALDH2* were identified using the TaqMan PCR method with TaqMan probes (*ADH1B*: rs1229984, *ALDH2*: rs671, Thermo Fisher Scientific, MA, USA), TaqMan genotyping master mix (Thermo Fisher Scientific), and a 7500 Fast real-time PCR system (Applied Biosystems, CA, USA).

### Endoscopic assessment and treatment

All upper gastrointestinal endoscopies were performed using conventional video endoscopes (GIF-Q260, GIF-Q260Z, GIF-H290, GIF-H290Z; Olympus Medical Systems, Tokyo, Japan). Board-certified endoscopists (Y.O., Y.S., Y.A., M.Y., and T.K.) of the Japanese Gastroenterological Endoscopy Society verified all endoscopic findings. We evaluated the esophageal LVLs by double-checking the endoscopic images that best reflect the degree of LVLs in the background esophageal mucosa. As previously reported^23^, the degree of LVLs in the esophageal mucosa was classified into three grades: grade A, no obvious LVLs; grade B, less than 10 LVLs per image; and grade C, more than 10 LVLs per image.

Superficial ESCCs were resected by endoscopic mucosal resection (EMR) or endoscopic submucosal dissection (ESD), which was performed in a standardized manner^54^ using conventional endoscopy (Q260J; Olympus Medical Systems, Tokyo, Japan). There was only one case of ESCC resected by EMR in the F-UR group, and there was no difference in the ratio of ESD to EMR in each group. The resected tissue generally contained a non-neoplastic margin of approximately 5 mm or more. The resection specimen diameters are shown in Supplementary Table 3. All endoscopically resected tissues were fixed in neutral-buffered 10% formalin solution immediately, paraffin-embedded, and diagnosed as ESCC or ESCN according to hematoxylin and eosin (HE) staining by two pathologists at Yamagata University Hospital.

### Cancer-related gene panel sequencing

Endoscopically resected tissue was sectioned at 2 mm intervals for pathological evaluation. The sectioned FFPE tissue from the endoscopically resected superficial ESCC and ESCN at a thickness of 5 μm was stretched on a polyethylene naphthalate-membrane slide (Leica Microsystems, Herborn, Germany). Using laser microdissection (LMD; Leica 6, Leica Microsystems), we dissected the FFPE tissues separately based on HE staining to distinguish between neoplastic and adjacent non-neoplastic epithelium. The stroma was not included in the dissected specimens after LMD. In addition, LMD was performed to cut out the non-neoplastic epithelium by carefully selecting the epithelium that was not damaged during endoscopic resection. DNA was extracted using the GeneRead DNA FFPE tissue kit (QIAGEN) according to the manufacturer’s protocol. DNA concentrations (Supp. Table 3) were measured using the Qubit dsDNA HS Assay Kit (Thermo Fisher Scientific).

The AmpliSeq library kit v2.0 (Thermo Fisher Scientific) was used for generating libraries according to the manufacturer's protocol. A total of 4 ng isolated DNA per sample was used as input. The libraries were quantified on a 2200 TapeStation system using high sensitivity D1000 reagents and high sensitivity D1000 screen tape (Agilent Technologies, CA, USA). Emulsion PCR was performed on the amplified libraries using the Ion OneTouch 2 system with Ion PI Template OT2 200 kit v3 (Thermo Fisher Scientific). Ion sphere particles were enriched using Ion OneTouch ES and loaded onto the Ion PI Chip v2. NGS was performed using Ion S5 with the Ion AmpliSeq Comprehensive Cancer Panel (Thermo Fisher Scientific), which targeted 409 genes (Suppl. Table 4). Read sequence files were run through the assembly programs constructed with Bowtie2 and BWA; subsequently, we ran a Freebayes program to obtain variants, which were detected using the two pipelines and annotated using ANNOVAR.

In the present study, we defined the detected variants as somatic variants predicted to be protein altering, after excluding synonymous variants; germline variants with a frequency of > 0.1% in the Genome Aggregation Database (https://gnomad.broadinstitute.org/) and the 1000 Genome database (https://www.internationalgenome.org/home); and germline variants with a frequency of > 1% in the cohort data for the in-house general healthy population (n = 176). The quality control for sequencing data and depth of coverage are provided in Supplementary Table 5.

### Immunohistochemical staining

FFPE slices were boiled in 10 nM sodium citrate buffer (pH 6.0) or Tris–EDTA buffer (pH 9.0) for 20 min under microwave irradiation for antigen retrieval. Immunohistochemical staining was performed using the polymeric method with the ImmPRESS universal PLUS polymer kit (Vector Laboratories, UK). After blocking with 2.5% normal horse serum for 20 min at 20 °C, the sliced tissues were incubated at 4 °C overnight with an anti-human p16INK4A monoclonal antibody (1:300; EPR1473, Abcam, UK) and anti-human p14ARF polyclonal antibody (1:200; E3X6D, Cell Signaling Technology, USA). The tissue was reacted with the ImmPRESS universal polymer reagent, containing the secondary antibody, for 30 min and then treated with diaminobenzidine for p16INK4A and p14ARF staining. More than 10% of the p16INK4A expression in the cytoplasm and p14ARF expression in the nucleus was considered positive in the neoplastic and adjacent non-neoplastic epithelium.

### Statistical analysis

Continuous and categorical variables were analyzed using the two-tailed Wilcoxon test and χ2 test, respectively. Multiple comparisons were performed using ANOVA, followed by the Tukey–Kramer test. *P*-values less than 0.05 were considered significant. Statistical calculations were performed using JMP 14.3.0 (SAS Institute, Japan, https://www.jmp.com/ja_jp/software/data-analysis-software.html) and R programming language version 3.6.1 (https://cran.r-project.org/bin/windows/base/old/3.6.1/). Statistical analysis for Fig. 1 was performed using R, and JMP was used for the other statistical analyses.

## Supplementary Information

Supplementary Information.

## Data Availability

All relevant original data are available from the corresponding author upon reasonable request.
