# Supplementary material for: Novel genomic alteration in superficial esophageal squamous cell neoplasms in non-smoker non-drinker females
Source: Sci Rep. 2021 Oct 11;11:20150. doi: 10.1038/s41598-021-99790-z (PMC8505482; doi:10.1038/s41598-021-99790-z)
Supplement: Supplementary file 1 — Supplementary Information. [file 41598_2021_99790_MOESM1_ESM.pdf]

Novel genomic alteration in superficial esophageal squamous cell neoplasms in non-smoker non-drinker females

Yusuke Onozato,[1] Yu Sasaki,[1,*] Yasuhiko Abe,[2] Hidenori Sato,[3] Makoto Yagi,[2] Naoko Mizumoto,[1] Takashi Kon,[1] Takayuki Sakai,[1] Minami Ito,[1] Matsuki Umehara,[1] Ayumi Koseki,[1] Yoshiyuki Ueno[1]

[1]Department of Gastroenterology, Faculty of Medicine, Yamagata University, 2-2-2 Iida-Nishi, Yamagata 990-9585, Japan

[2]Division of Endoscopy, Yamagata University Hospital, 2-2-2 Iida-Nishi, Yamagata 990-9585, Japan

[3]Genomic Information Analysis Unit, Department of Genomic Cohort Research, Faculty of Medicine, Yamagata University

***Correspondence**:

Yu Sasaki

Department of Gastroenterology, Faculty of Medicine, Yamagata University,

2-2-2 Iida-Nishi, Yamagata 990-9585, Japan

E-mail: y-sasaki@med.id.yamagata-u.ac.jp

Tel: +81-23-628-5309 Fax: +81-23-628-5311

Supplementary information accompanying this paper

Supplementary figure 1. Total number of somatic variants and frequency of the types in neoplastic epithelium

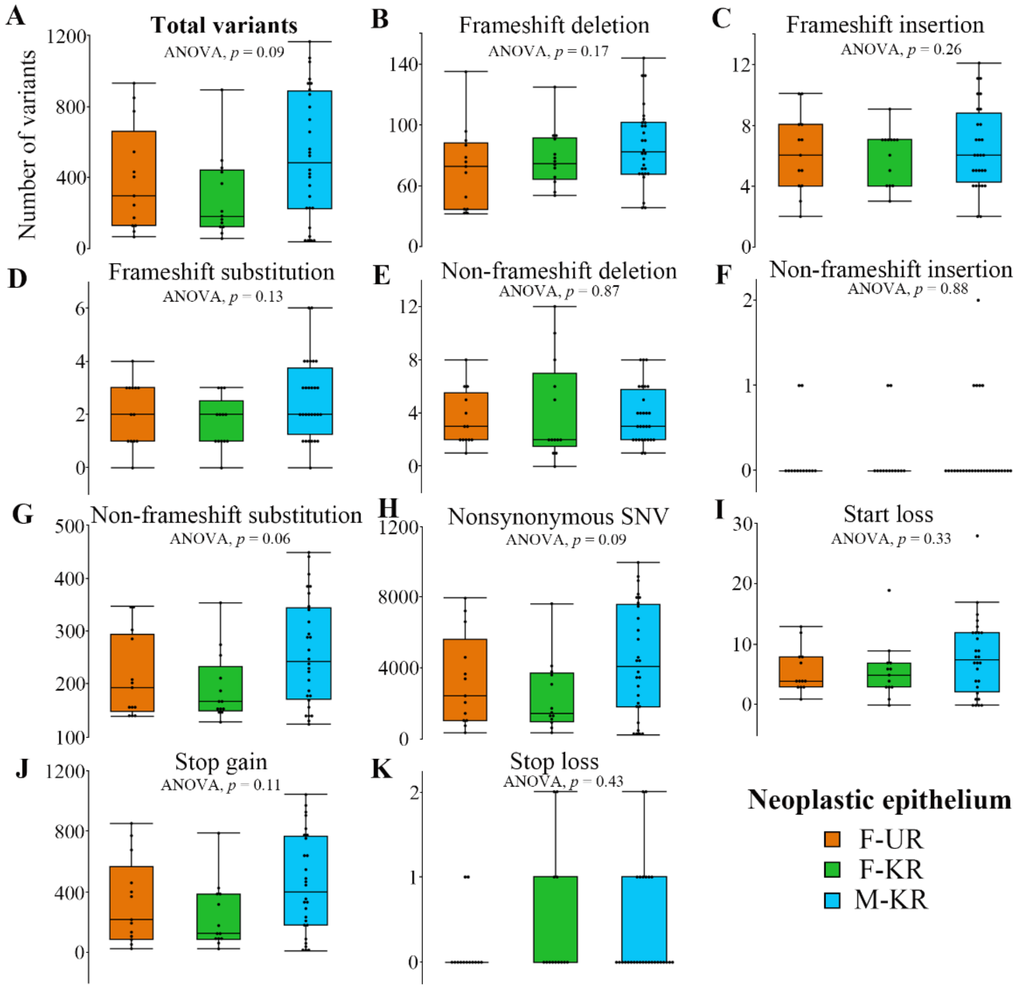

There were no significant differences in the total number of somatic variants (A) and the frequency of frameshift deletion (B), frameshift insertion (C), frameshift substitution (D), non-frameshift deletion (E), non-frameshift (F), non-frameshift substitution (G), nonsynonymous single nucleotide variant (H), start loss (I), stop gain (J), and stop loss (K) in neoplastic mucosa among the three groups.

Box plot: the bottom and top of each box represent the 25th and 75th percentiles,

respectively, and the band in the box is the median. Whiskers: the lowest datum is within the minimum, and the highest datum is still within the 1.5 IQR of the upper quartile. We used ANOVA with a post hoc Tukey–Kramer test to evaluate the statistical differences among the groups. F-UR, female unknown-risk; F-KR, female known-risk; M-KR, male known-risk; ANOVA, analysis of variance; IQR, interquartile range.

Supplementary figure 2. Total number of somatic variants and frequency of the types in adjacent non-neoplastic epithelium

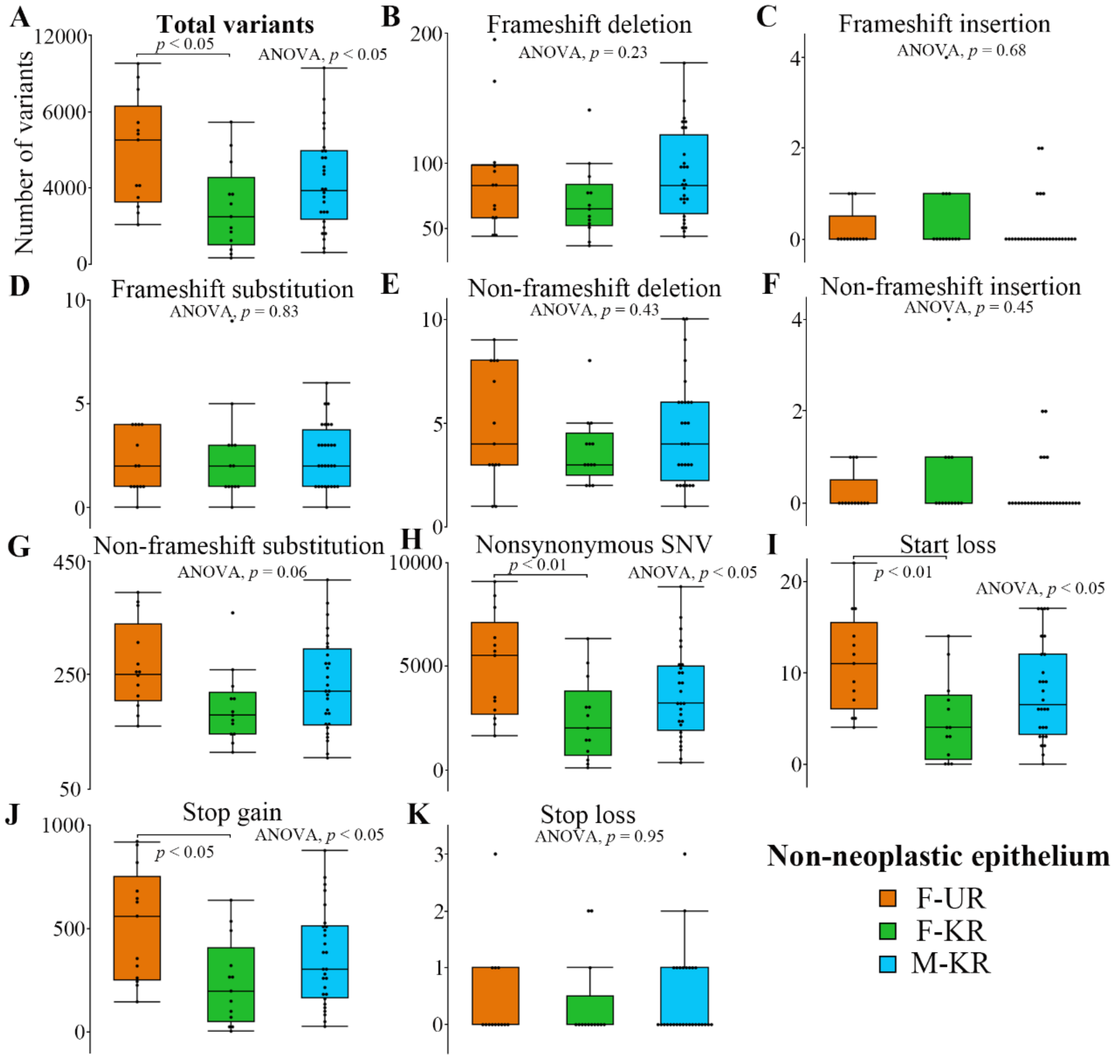

The total number of somatic variants (A) and the frequency of nonsynonymous single nucleotide variants (H), start loss (I), and stop gain (J) were significantly higher in the F-UR group than in the F-KR group, whereas there was no difference in the frequency of frameshift deletion (B), frameshift insertion (C), frameshift substitution (D), non-frameshift deletion (E), non-frameshift insertion (F), non-frameshift substitution (G), and stop loss (K).

Box plot: the bottom and top of each box represent the 25th and 75th percentiles, respectively, and the band in the box is the median. Whiskers: the lowest datum is within the minimum, and the highest datum is still within the 1.5 IQR of the upper quartile. We used ANOVA with a post hoc Tukey–Kramer test to evaluate the statistical differences between groups. F-UR, female unknown-risk; F-KR, female known-risk; M-KR, male known-risk; ANOVA, analysis of variance; IQR, interquartile range.

Supplementary figure 3. Variant allele frequency of the *CDKN2A*, *CDKN2B*, *CDKN2C*, and *TP53* genes

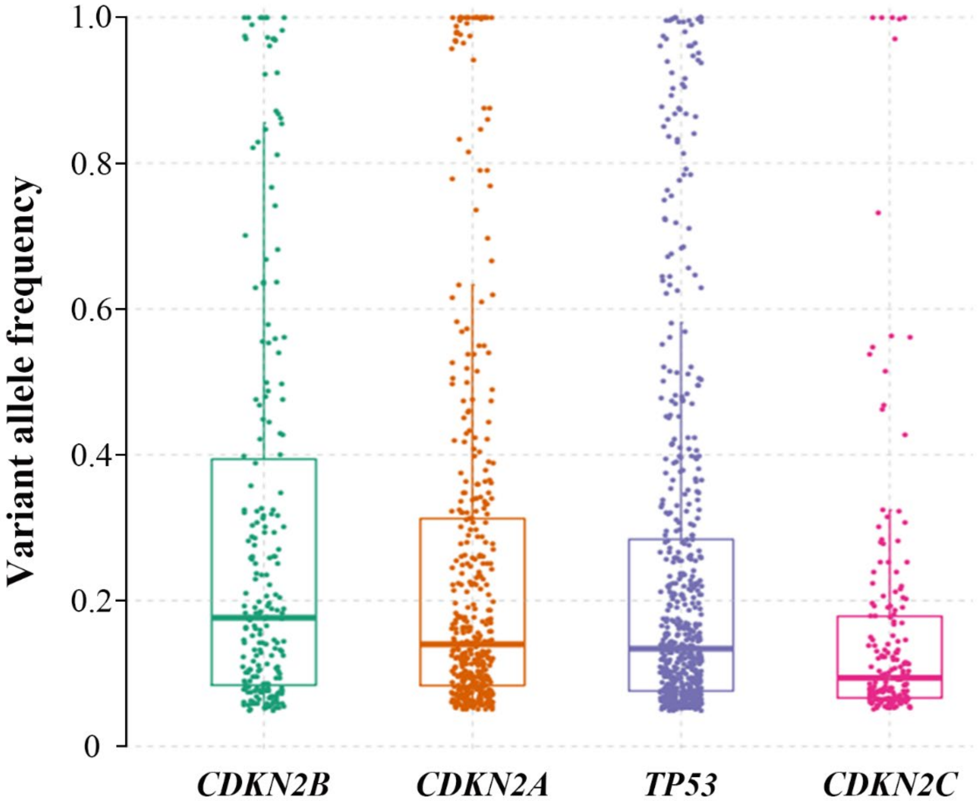

We assessed the somatic variant allele frequencies for the *CDKN2A* gene, including its gene families *CDKN2B* and *CDKN2C*. High frequencies of variants above 0.9 were observed for *CDKN2A*, as well as *TP53*, which exhibited the highest variant frequency in the ESCC.

Box plot: the bottom and top of each box represent the 25th and 75th percentiles, respectively, and the band in the box is the median. Whiskers: the lowest datum is within the minimum, and the highest datum is still within the 1.5 IQR of the upper quartile. We used ANOVA with a post hoc Tukey–Kramer test to evaluate the statistical differences among the groups. *CDKN2A,* cyclin-dependent kinase inhibitor 2A; *CDKN2B,* cyclin-

dependent kinase inhibitor 2B; *CDKN2C*, *cyclin-dependent kinase inhibitor 2C*; ESCC, esophageal squamous cell carcinoma; *TP53*, tumor protein 53; ANOVA, analysis of variance; IQR, interquartile range.

Supplementary figure 4. Correlation between pathological type and somatic variant frequency of the *CDKN2A* gene

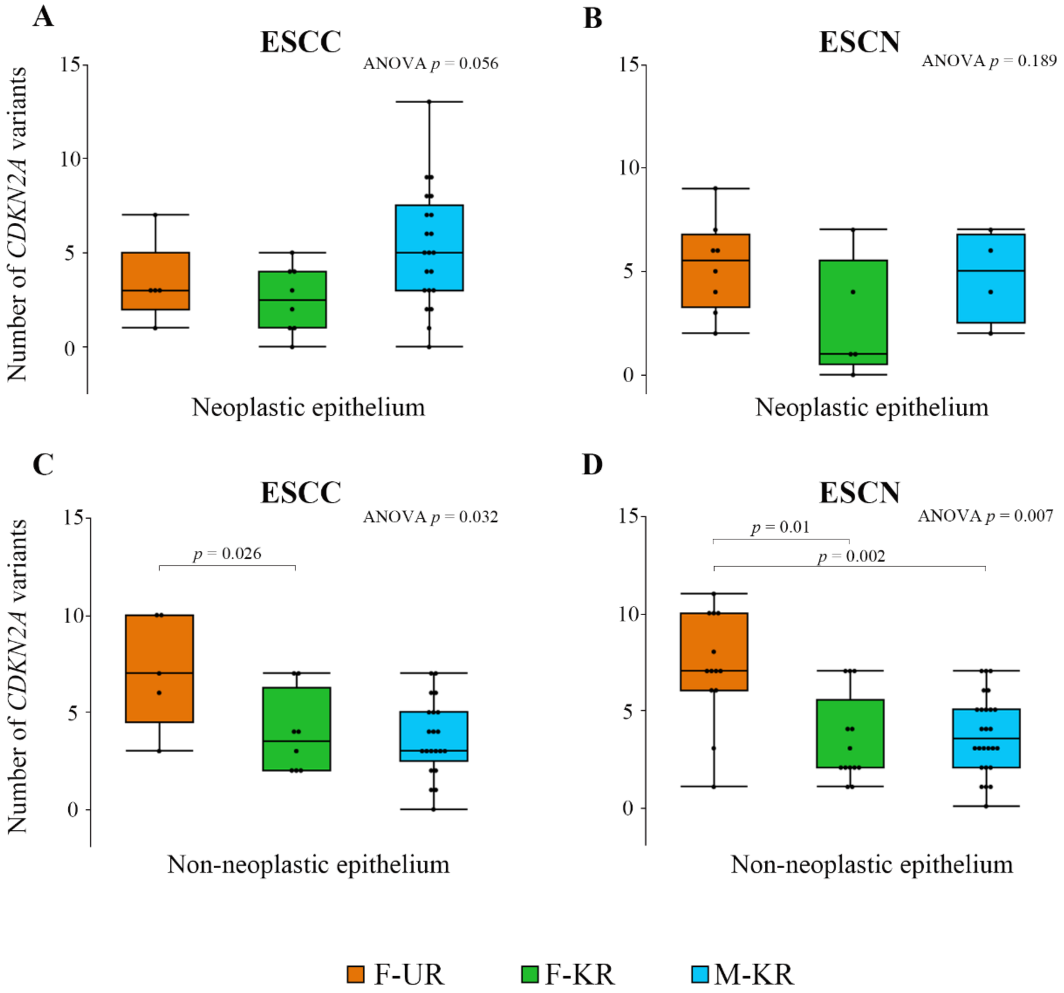

The somatic variant frequency for the *CDKN2A* gene in neoplastic and non-neoplastic epithelium between the three groups was compared between ESCC and ESCN patients. In the neoplastic mucosa, no significant difference was observed in the variant frequency among the three groups in both ESCC (a) and ESCN (b) patients. In the non-neoplastic epithelium, the variant frequency was higher in F-UR than in F-KR and M-KR in both ESCC ($p = 0.032$) and ESCN patients ($p = 0.007$).

Box plot: the bottom and top of each box represent the 25th and 75th percentiles, respectively, and the band in the box is the median. Whiskers: the lowest datum is within the minimum, and the highest datum is still within the 1.5 IQR of the upper quartile. We used ANOVA with a post hoc Tukey–Kramer test to evaluate the statistical differences among the groups. *CDKN2A,* cyclin-dependent kinase inhibitor 2A; ESCC, esophageal squamous cell carcinoma; ESCN, esophageal squamous cell neoplasia; ANOVA, analysis of variance; IQR, interquartile range.

Supplementary figure 5. Correlation between age and somatic variant frequency of the *CDKN2A* gene

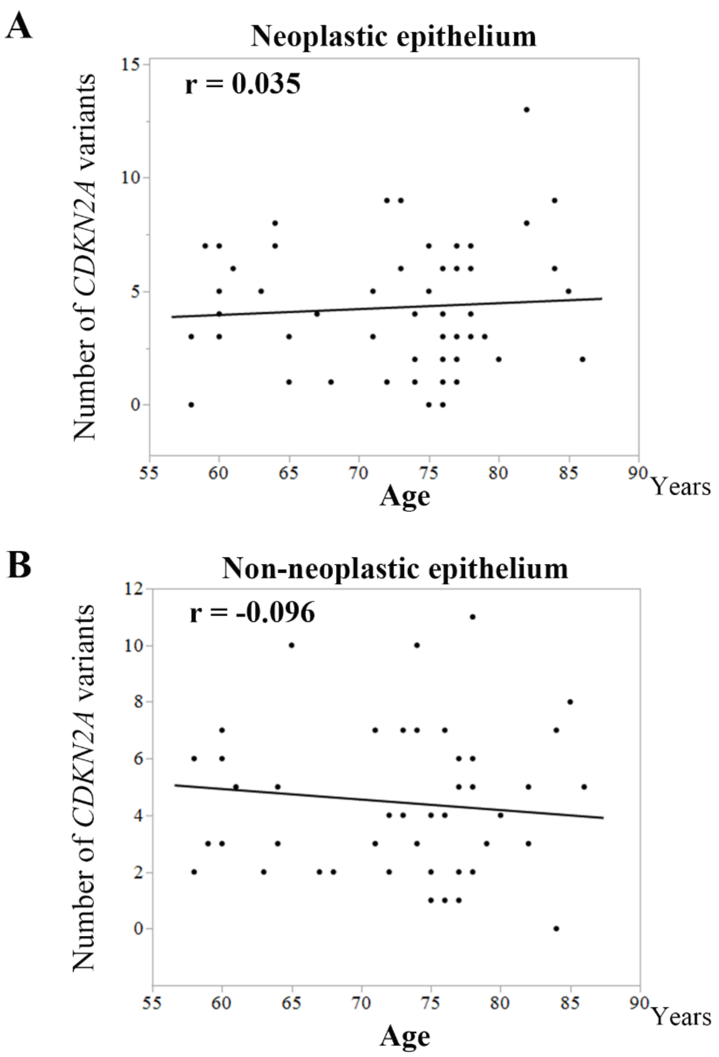

The correlation between somatic variant frequency of *CDKN2A* gene and age was assessed using Spearman's rank correlation coefficient. There was no significant correlation between the variant and age in either the neoplastic (a) or non-neoplastic (b) epithelium. *CDKN2A,* cyclin-dependent kinase inhibitor 2A.

Supplementary figure 6. Copy number alterations of the region harboring the *CDKN2A* loci

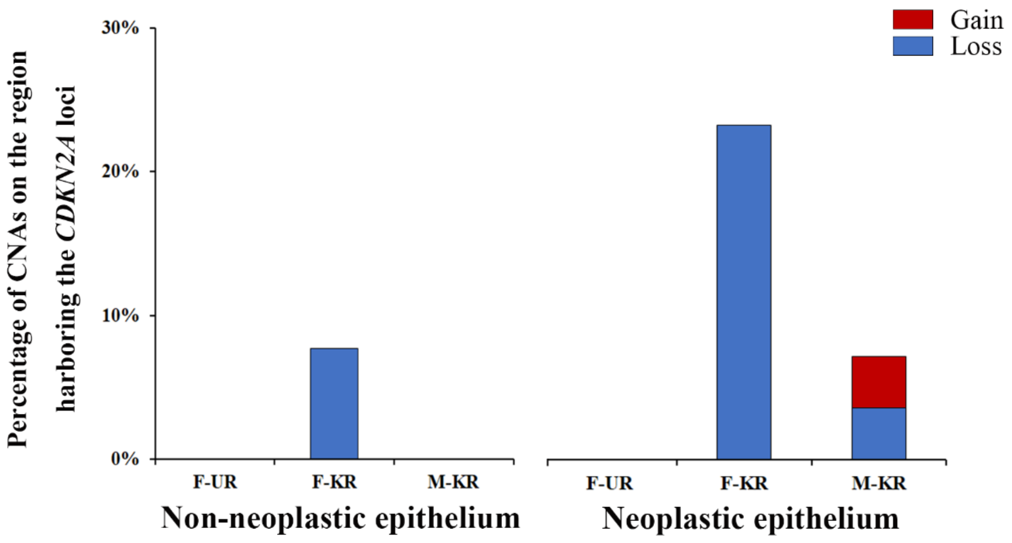

The frequencies of CNAs of the region harboring the *CDKN2A* loci in non-neoplastic and neoplastic epithelium were compared among the three groups. Overall, CNAs were detected in 11.3% (6 of 53) of the patients. In the non-neoplastic epithelium, CNAs were detected in one patient in the F-KR (7.7%), but not in the M-KR and F-UR groups (p = 0.23). In the neoplastic epithelium, CNAs were detected in 3 patients in the F-KR (23.1%) and 2 patients in the M-KR group (7.4%), but not in the F-UR groups (p = 0.13). There were no patients with the loss of heterozygosity in either the non-neoplastic or neoplastic epithelium. CNAs, copy number alterations.

Supplementary table 1. Comparison of the *ADH1B* and *ALDH2* allele types of the patients in the present study

		Total $n = 148$	Female unknown-risk $n = 15$ (10.1)	Female known-risk $n = 17$ (11.5)	Male known-risk $n = 116$ (78.4)	p-value[a]
ADH1B	*1/1, *n* (%)	16 (10.8)	0 (0)	3 (17.6)	13 (11.2)	0.002
	*1/2, *n* (%)	29 (19.6)	0 (0)	2 (11.8)	27 (23.3)	
	*2/2, *n* (%)	58 (39.2)	11 (73.3)	5 (29.4)	42 (36.2)	
	Unknown, *n* (%)	45 (30.4)	4 (26.7)	7 (41.2)	34 (29.3)	
ALDH2	*1/1, *n* (%)	52 (35.1)	4 (26.7)	6 (35.3)	42 (36.2)	0.467
	*1/2, *n* (%)	51 (34.5)	7 (46.6)	4 (23.5)	40 (34.5)	
	*2/2, *n* (%)	0 (0)	0 (0)	0 (0)	0 (0)	
	Unknown, *n* (%)	45 (30.4)	4 (26.7)	7 (41.2)	34 (29.3)	

Values are expressed as the median (IQR) or number (%). Alleles of *ADH1B* and *ALDH2* were available in subjects with preserved whole blood (*n* = 106, 84 males and 22 females). [a]Comparisons among the three groups were performed using the $\chi 2$ test.

ADH1B, alcohol dehydrogenase 1B; *ALDH2*, aldehyde dehydrogenase-2; IQR, interquartile range.

Supplementary table 2. Comparison of the *ADH1B* and *ALDH2* allele types of the patients who underwent NGS analysis in the present study

		Total<br>$n = 53$	Female unknown-risk<br>$n = 13$	Female known-risk<br>$n = 13$	Male known-risk<br>$n = 27$	p-value[a]
ADH1B	*1/1, *n* (%)	5 (9.4)	0 (0)	2 (15.4)	3 (11.1)	0.049
	*1/2, *n* (%)	9 (17.0)	0 (0)	2 (15.4)	7 (25.9)	
	*2/2, *n* (%)	22 (41.5)	10 (76.9)	4 (30.8)	8 (29.6)	
	Unknown, *n* (%)	17 (32.1)	3 (23.1)	5 (38.5)	9 (33.3)	
ALDH2	*1/1, *n* (%)	19 (35.8)	4 (30.8)	5 (38.5)	10 (37.0)	0.602
	*1/2, *n* (%)	17 (32.1)	6 (46.2)	3 (23.0)	8 (29.6)	
	*2/2, *n* (%)	0 (0)	0 (0)	0 (0)	0 (0)	
	Unknown, *n* (%)	17 (32.0)	3 (23.0)	5 (38.5)	9 (33.3)	

Values are expressed as median (IQR) or number (%). [a]Comparisons among the three groups were performed using the $\chi 2$ test.

ADH1B, alcohol dehydrogenase 1B; *ALDH2*, aldehyde dehydrogenase-2; IQR, interquartile range.

Supplementary table 3. Tissue area resected by laser microdissection and the concentration of isolated DNA

	Total<br>*n* = 53	Female unknown-risk<br>*n* = 13	Female known-risk<br>*n* = 13	Male known-risk<br>*n* = 27	*p*-value[a]
**Endoscopically resected specimen**, mm					
Major axis	29 (20–38)	25 (17.5–32)	25 (18.5–30)	33 (25–40)	0.061
Minor axis	21 (15–26.5)	15 (10–22.5)	20 (15–25)	25 (17–30)	0.064
**Area cut out using LMD**, mm^2					
Non-neoplastic epithelium	1.51 (1.08–2.16)	1.34 (0.94–2.37)	1.21 (0.99–1.97)	1.52 (1.19–2.36)	0.626
Neoplastic epithelium	1.07 (0.78–2.30)	1.51 (0.50–6.55)	0.89 (0.59–1.70)	1.65 (0.84–2.42)	0.252
**DNA concentration isolated from tissue cut out using LMD**, ng/μL					
Non-neoplastic epithelium	0.05 (0.04–0.07)	0.05 (0.04–0.10)	0.06 (0.03–0.08)	0.04 (0.03–0.06)	0.089
Neoplastic epithelium	0.09 (0.05–0.23)	0.19 (0.07–0.53)	0.06 (0.03–0.17)	0.08 (0.05–0.30)	0.053

Values are expressed as median (IQR) or number (%). [a]Comparisons among the three groups were performed using ANOVA.

LMD, laser microdissection.

Supplementary table 4. Target genes in the Ion AmpliSeq Comprehensive Cancer Panel

ABL1	*BAI3*	*CARD11*	*DAXX*	*EGFR*	*FAM123B*	*G6PD*	*HCAR1*	*ICK*	*JAK1*	*KAT6A*	*LAMP1*	*MAF*	*NBN*	*PAK3*	*RAD50*	*SAMD9*	*TAF1*	*UBR5*	*VHL*	*WAS*	*XPA*	*ZNF384*
ABL2	*BAP1*	*CASC5*	*DCC*	*EML4*	*FANCA*	*GATA1*	*HIF1A*	*IDH1*	*JAK2*	*KAT6B*	*LCK*	*MAFB*	*NCOA1*	*PALB2*	*RAF1*	*SBDS*	*TAF1L*	*UGT1A1*		*WHSC1*	*XPC*	*ZNF521*
ACVR2A	*BCL10*	*CBL*	*DDB2*	*EP300*	*FANCC*	*GATA2*	*HLF*	*IDH2*	*JAK3*	*KDM5C*	*LIFR*	*MAGEA1*	*NCOA2*	*PARP1*	*RALGDS*	*SDHA*	*TAL1*	*USP9X*		*WRN*	*XPO1*	
ADAMTS20	*BCL11A*	*CCND1*	*DDIT3*	*EP400*	*FANCD2*	*GATA3*	*HNF1A*	*IGF1R*	*JUN*	*KDM6A*	*LPHN3*	*MAGI1*	*NCOA4*	*PAX3*	*RARA*	*SDHB*	*TBX22*			*WT1*	*XRCC2*	
AFF1	*BCL11B*	*CCND2*	*DDR2*	*EPHA3*	*FANCF*	*GDNF*	*HOOK3*	*IGF2*		*KDR*	*POT1*	*MALT1*	*NF1*	*PAX5*	*RB1*	*SDHC*	*TCF12*					
AFF3	*BCL2*	*CCNE1*	*DEK*	*EPHA7*	*FANCG*	*GNA11*	*HRAS*	*IGF2R*		*KEAP1*	*LPP*	*MAML2*	*NF2*	*PAX7*	*RECQL4*	*SDHD*	*TCF3*					
AKAP9	*BCL2L1*	*CD79A*	*DICER1*	*EPHB1*	*FAS*	*GNAQ*	*HSP90AA1*	*IKBKB*		*KIT*	*LRP1B*	*MAP2K1*	*NFE2L2*	*PAX8*	*REL*	*SEPT9*	*TCF7L1*					
AKT1	*BCL2L2*	*CD79B*	*DNMT3A*	*EPHB4*	*FBXW7*	*GNAS*	*HSP90AB1*	*IKBKE*		*KLF6*	*LTF*	*MAP2K2*	*NFKB1*	*PBRM1*	*RET*	*SETD2*	*TCF7L2*					
AKT2	*BCL3*	*CDC73*	*DPYD*	*EPHB6*	*FGFR1*	*GPR124*		*IKZF1*		*KRAS*	*LTK*	*MAP2K4*	*NFKB2*	*PBX1*	*RHOH*	*SF3B1*	*TCL1A*					
AKT3	*BCL6*	*CDH1*	*DST*	*ERBB2*	*FGFR2*	*GRM8*		*IL2*				*MAP3K7*	*NIN*	*PDE4DIP*	*RNASEL*	*SGK1*	*TET1*					
ALK	*BCL9*	*CDH11*		*ERBB3*	*FGFR3*	*GUCY1A2*		*IL21R*				*MAPK1*	*NKX2-1*	*PDGFB*	*RNF2*	*SH2D1A*	*TET2*					
APC	*BCR*	*CDH2*		*ERBB4*	*FGFR4*			*IL6ST*				*MAPK8*	*NLRP1*	*PDGFRA*	*RNF213*	*SMAD2*	*TFE3*					
AR	*BIRC2*	*CDH20*		*ERCC1*	*FH*			*IL7R*				*MARK1*	*NOTCH1*	*PDGFRB*	*ROS1*	*SMAD4*	*TGFBR2*					
ARID1A	*BIRC3*	*CDH5*		*ERCC2*	*FLCN*			*ING4*				*MARK4*	*NOTCH2*	*PER1*	*RPS6KA2*	*SMARCA4*	*TGM7*					
ARID2	*BIRC5*	*CDK12*		*ERCC3*	*FLI1*			*IRF4*				*MBD1*	*NOTCH4*	*PGAP3*	*RRM1*	*SMARCB1*	*THBS1*					
ARNT	*BLM*	*CDK4*		*ERCC4*	*FLT1*			*IRS2*				*MCL1*	*NPM1*	*PHOX2B*	*RUNX1*	*SMO*	*TIMP3*					
ASXL1	*BLNK*	*CDK6*		*ERCC5*	*FLT3*			*ITGA10*				*MDM2*	*NRAS*	*PIK3C2B*	*RUNX1T1*	*SMUG1*	*TLR4*					
ATF1	*BMPR1A*	*CDK8*		*ERG*	*FLT4*			*ITGA9*				*MDM4*	*NSD1*	*PIK3CA*		*SOCS1*	*TLX1*					
ATM	*BRAF*	*CDKN2A*		*ESR1*	*FN1*			*ITGB2*				*MEN1*	*NTRK1*	*PIK3CB*		*SOX11*	*TNFAIP3*					
ATR	*BRD3*	*CDKN2B*		*ETS1*	*FOXL2*			*ITGB3*				*MET*	*NTRK3*	*PIK3CD*		*SOX2*	*TNFRSF14*					
ATRX	*BRIP1*	*CDKN2C*		*ETV1*	*FOXO1*							*MITF*	*NUMA1*	*PIK3CG*		*SRC*	*TNK2*					
AURKA	*BTK*	*CEBPA*		*ETV4*	*FOXO3*							*MLH1*	*NUP214*	*PIK3R1*		*SSX1*	*TOP1*					
AURKB	*BUB1B*	*CHEK1*		*EXT1*	*FOXP1*							*MLL*	*NUP98*	*PIK3R2*		*STK11*	*TP53*					
AURKC		*CHEK2*		*EXT2*	*FOXP4*							*MLL2*		*PIM1*		*STK36*	*TPR*					
AXL		*CIC*		*EZH2*	*FZR1*							*MLL3*		*PKHD1*		*SUFU*	*TRIM24*					
		CKS1B										*MLLT10*		*PLAG1*		*SYK*	*TRIM33*					
		CMPK1										*MMP2*		*PLCG1*		*SYNE1*	*TRIP11*					
		COL1A1										*MN1*		*PLEKHG5*			*TRRAP*					
		CRBN										*MPL*		*PML*			*TSC1*					
		CREB1										*MRE11A*		*PMS1*			*TSC2*					
		CREBBP										*MSH2*		*PMS2*			*TSHR*					
		CRKL										*MSH6*		*POU5F1*								
		CRTC1										*MTOR*		*PPARG*								
		CSF1R										*MTR*		*PPP2R1A*								
		CSMD3										*MTRR*		*PRDM1*								
		CTNNA1										*MUC1*		*PRKAR1A*								
		CTNNB1										*MUTYH*		*PRKDC*								
		CYLD										*MYB*		*PSIP1*								
		CYP2C19										*MYC*		*PTCH1*								
		CYP2D6										*MYCL1*		*PTEN*								
												MYCN		*PTGS2*								
												MYD88		*PTPN11*								
												MYH11		*PTPRD*								
												MYH9		*PTPRT*								

Supplementary table 5. Quality control of sequencing data and depth of coverage.

	Median (IQR)
**Quality Control**	
Mapping rate, %	98.4 (95.3–99.5)
Error rate, %	0.19 (0.11–0.26)
Indel rate, %	0.36 (0.35–0.39)
Mean read, bp	107 (106–109)
Strand bias	0.49 (0.49–0.50)
Maximum base QC	26 (24–31)
**Depth of coverage**	392 (313–498)
